# The second touch hypothesis: T cell activation, homing and polarization

**DOI:** 10.12688/f1000research.3-37.v2

**Published:** 2014-08-04

**Authors:** Klaus Ley

**Affiliations:** 1 Division of Inflammation Biology, La Jolla Institute for Allergy & Immunology, La Jolla, CA 92037, USA

## Abstract

The second touch hypothesis states that T cell activation, proliferation, induction of homing receptors and polarization are distinguishable and, at least in part, sequential. The second touch hypothesis maintains that full T cell polarization requires T cell interaction with antigen-presenting cells (DCs, macrophages, B cells and certain activated stromal cells) in the non-lymphoid tissue where the antigen resides. Upon initial antigen encounter in peripheral lymph nodes (PLN), T cells become activated, proliferate and express homing receptors that enable them to recirculate to the (inflamed) tissue that contains the antigen. Differentiation into the T helper lineages Th1, Th2, Th17 and induced regulatory T cells (iTreg) requires additional antigen presentation by tissue macrophages and other antigen presenting cells (APCs) in the inflamed tissue. Here, I present a conceptual framework for the importance of peripheral (non-lymphoid) antigen presentation to antigen-experienced T cells.

## The role of innate immune cells

Most animals (including all invertebrates) do not have adaptive immune systems, yet are well protected from pathogens. Yet all these animals have macrophages or macrophage-like cells. These macrophages directly sense pathogens and mount an appropriate defensive response. In vertebrates with an adaptive immune system, macrophages (and other innate immune cells) drive the polarization of the adaptive immune response. This was first shown in mice: certain inbred mouse strains have a Th1 bias (like C57BL/6) and others have a Th2 bias (like Balb/c). The underlying reason for this bias is not in the T cell compartment, but is found in the innate immune system ^1^. This was shown by using immunodeficient mice that do not have an adaptive immune system, yet still preserve the Th1/Th2 bias. This finding gave rise to the concept of M1 and M2 macrophages ^1^, where M1 macrophages metabolize arginine to nitric oxide (NO) and citrulline through iNOS and M2 macrophages metabolize arginine to ornithine and urea through arginase. NO promotes bacterial killing and inflammation, whereas ornithine promotes wound healing and collagen biosynthesis.

M2 macrophages produce TGF-β, which is secreted in an inactive form that requires activation by a process that depends on αVβ8 integrin ^2– 4^. The activating integrin must be expressed on the same cell that presents the antigen, suggesting that only TGF-β secreted by αVβ8 integrin-expressing macrophages and DCs is relevant for iTreg induction.

## Macrophage activation

Macrophages express a large number of cell surface and cytosolic receptors that allow them to recognize bacteria, fungi, parasites, viruses, but also altered self molecules. The macrophage activating receptors fall into five families:

- Toll-like receptors (TLRs)
- NOD-like receptors (NLRs), which are central to the assembly of the inflammasome and production of the IL-1 and IL-18 family of inflammatory cytokines
- RIG-I like receptors (RLRs)
- C-type lectins, which includes receptors for fungal pathogens like dectin-1
- Scavenger receptors, including scavenger receptor-A, B and CD36

Engagement of many of these receptors activates members of the transcription factor family NF-kB, which in turn is responsible for production of many inflammatory cytokines. How the engagement of specific pattern recognition receptors is related to the cytokines that drive CD4 T cell differentiation is an area of active investigation. Macrophages can secrete IL-1α and β, IL-18, IL-6, IL-12, IL-23, α and β interferons, IL-4, IL-10 and TGF-β. Activated macrophages express a range of molecules from the TNF and TNF receptor superfamilies.

## Second touch hypothesis

**Figure 1.**
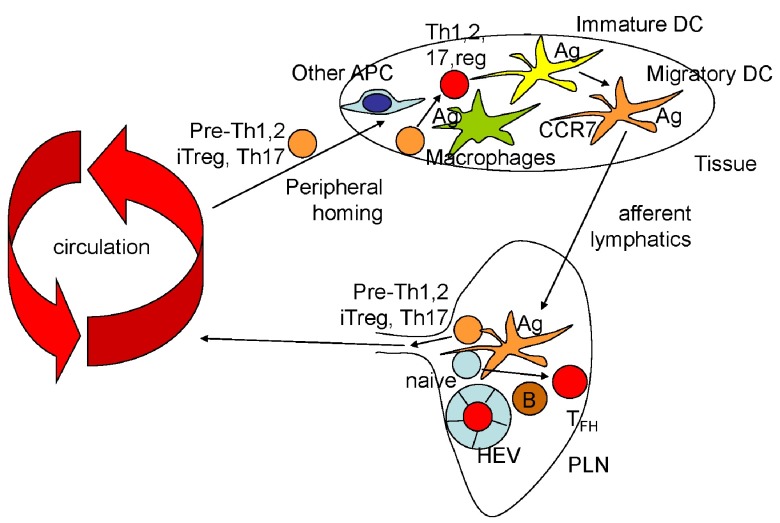
The second touch hypothesis. Naïve T cells (light blue) enter peripheral lymph nodes (PLN) through high endothelial venules (HEV), where they encounter antigen (Ag) presented by a migratory dendritic cell (DC) or by a PLN-resident B cell (brown). If signals induce Bcl6 and CXCR5, the T cells may enter the germinal center and become follicular helper T cells (TFH, red). Upon encountering antigen in the context of co-stimulatory molecules like CD80 and CD86, most T cells will express homing receptors, downregulate the sphingosine-1-phosphate receptor S1P1 and leave the PLN. These cells (orange) are only partially programmed and referred to as pre-Th1, pre-Th2, pre-Th17 and pre-iTreg. These cells circulate and reach various tissues, including the inflamed tissue from which the antigen came. There, they encounter DCs, macrophages and other antigen-presenting cells (APCs) such as endothelial cells. These cells present antigen in the context of different co-stimulatory molecules such as TNFSF members, and in the context of inflammatory cytokines found in the inflamed tissue. If the prevailing signal is IL-12, the T cell will commit to Th1, if IL-4, 5 and 13, to Th2, if IL-1, TGF-β, IL-6, IL-21, to Th17. If the cytokine environment is dominated by TGF-β, an M2 cytokine, the T cell will become an iTreg.

The second touch hypothesis as formulated here states that T cell activation, proliferation, induction of homing receptors, and polarization are distinguishable and at least in part sequential. The second touch hypothesis maintains that full CD4 T cell polarization requires CD4 T cell interaction with antigen-presenting cells (DCs, macrophages, B cells and certain activated stromal cells) in the inflamed non-lymphoid tissue where the antigen was first encountered ( Figure 1).

This hypothesis holds that homing receptors are induced first, soon after activation of naïve T cells. Expression of homing receptors capacitates these T cells to leave the lymphatic system and enter specific tissues, including those where the antigen was first encountered.

**Figure 2.**
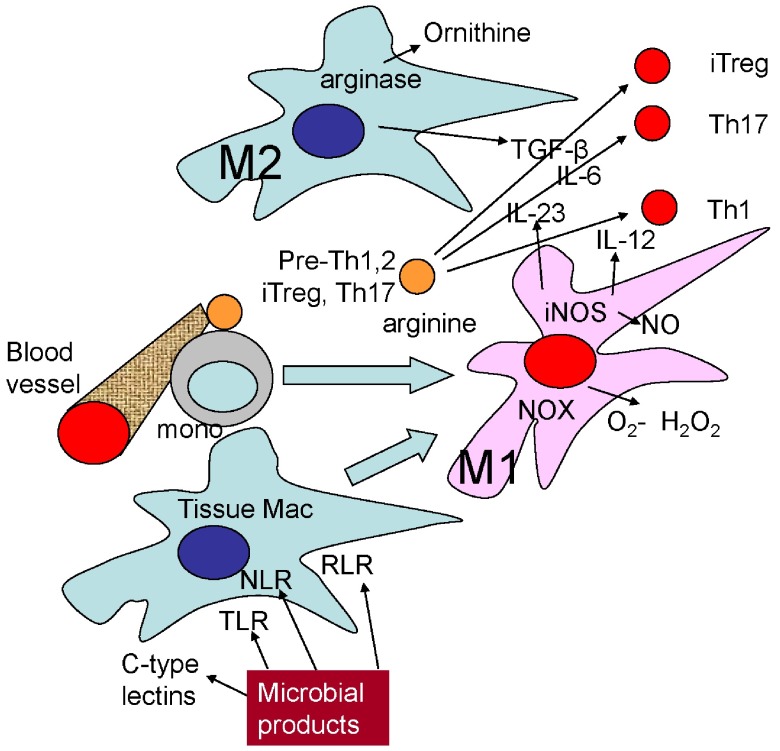
Pathogen sensors and cytokine environment. Tissue macrophages have a natural bias toward M2 (express arginase, produce TGF-β). T cells in this environment are likely to commit to the iTreg lineage. If tissue macrophages sense the presence of pathogens through their TLRs, NLRs, RLRs (see definitions in text) or C-type lectin receptors, they convert to M1 (express iNOS, produce IL-12, IL-23, upregulate MHC-II and CD86). M1 differentiation also induces expression of chemokines that attract more monocytes from the circulation, which have a propensity to become M1-polarized after transmigration. T cells receiving a second touch in this environment are likely to commit to Th1 if IL-12 dominates or Th17 if IL-23 dominates. The tissue environment producing Th2 cells is not well understood, but may involve M2 macrophages.

The second touch hypothesis also holds that antigen-experienced T cells require a “second touch” by seeing antigen presented on a tissue macrophage or DC in the context of co-stimulatory molecules and in the inflammatory cytokine environment of the inflamed tissue ( Figure 2). This “second touch” appears to be required for full differentiation to Th1, Th2, Th17, Treg or TFH. This may involve chromatin remodeling of the loci needed for the respective differentiation (see below).

Naïve αβ T cells are activated by contacting antigen-presenting cells in secondary lymphoid organs ^5^. Dendritic cells (DCs) are the most effective antigen-presenting cells for naïve T cells. T cells must receive two signals, one from T cell receptor (TCR) engagement with antigenic peptide in the context of MHC-II for CD4 or MHC-I for CD8 T cells, and the other one through CD28 engagement by CD80 and/or CD86 ^6^. Both the MHC and co-stimulatory signals are provided by the same dendritic cell ^7^. The cytokine milieu at the time of antigen presentation drives T cell polarization. Here, I will only consider helper (CD4) T cells, but similar lineages, subsets and polarizations have also been described for CD8 T cells.

- Th1 develop in response to IL-12 and IFN-γ, express the defining transcription factor T-bet ( *Tbx21*) and secrete the signature cytokine IFN-γ
- Th2 develop in response to IL-4, IL-5 and IL-13, express the defining transcription factor GATA-3 ( *Gata3*) and secrete the signature cytokines IL-4, IL-5 and IL-13
- iTreg develop in response to TGF-β, express the defining transcription factor FOXP3 ( *Foxp3*) and secrete the signature cytokine IL-10
- Th17 develop in response to IL-6, TGF-β and IL-1, express the defining transcription factor ROR-γt ( *Rorc*) and secrete the signature cytokines IL-17A, IL-17F and IL-21
- Follicular helper T cells (TFH) develop in response to CD40 and ICOS ligand, express the defining transcription factor BCL6 ( *Bcl6*) and secrete the signature cytokine IL-21

In the traditional view, naïve T cell activation, proliferation and differentiation are all considered as concomitant, simultaneous processes. The second touch hypothesis holds that full differentiation is only achieved after these T cells return to the site of inflammation and undergo a recall response.

## Antigen transport to lymph nodes

Antigenic proteins reach draining lymph nodes either as soluble antigens or by way of migratory DCs that acquired antigen in the inflamed tissue. Soluble antigen is presented by at least 3 types of lymph node-resident DCs to naïve T cells. Multiple short (3 minutes) interactions allow the T cell to become activated. A little later, migratory DCs enter the draining lymph node through afferent lymphatics and present higher doses of antigen, or more highly processed antigen, to naïve T cells, resulting in long-term (~50 min) interactions ^6^. Whether these longer-term interactions activate T cells more fully than repeated 3 min interactions is unclear. Many, if not all, migratory DCs are monocyte-derived, and only migrate to the regional lymph nodes under conditions of inflammation. These DCs must undergo a “maturation”, which involves expression of the chemokine receptor CCR7, and upregulation of the co-stimulatory molecule CD86. Maturation can be achieved by TLR ligands such as LPS or by engagement of CD40. Only mature DCs acquire the ability to migrate to lymph nodes ^8^. Mature DCs share phenotypic characteristics and gene expression with M1 macrophages. M1 macrophages are characterized by the expression of iNOS, producing NO from arginine ^1^. M2 macrophages express arginase, an enzyme that converts arginine to ornithine. The environment (M1, M2) during antigen encounter is likely to influence the ensuing T cell response.

## T cell activation

Naïve T cell activation in peripheral lymph nodes is a dramatic and violent process ^9^. Most activated T cells die by apoptosis in the lymph node where they were generated. The resulting apoptotic bodies are phagocytosed by local macrophages, triggering an anti-inflammatory signal ^10, 11^ that induces iTregs ^12^. iTregs are a natural by-product of any ongoing immune response, whether in infections or autoimmune diseases ^13^. Some activated T cells survive and acquire activation markers including:

- Expression of CD69 (which inhibits the sphingosine-1-phosphate receptor S1P1 ^14^ and helps to keep the activated T cell from recirculating)
- Expression of CD25 (a subunit of the IL-2 receptor)
- Production and secretion of IL-2
- Expression of CD44, a hyaluronic acid receptor
- Downregulation of CD62L (L-selectin)

Antigen-experienced T cells proliferate in the lymph node for 3–7 days and acquire peripheral homing receptors that allow them to return to the tissue where the antigen resides.

## Homing receptors

Homing receptors are defined as cell surface molecules that allow T cells to attach to endothelial cells in specific tissues and organs, transmigrate through the endothelial cell monolayer and the basement membrane and infiltrate the inflamed tissues. One category of homing receptors are chemokine receptors; heptahelical cell surface receptors that are coupled through heterotrimeric G-proteins. These include:

- CXCR3 and CCR5 in Th1 cells
- CCR4 in Th2 cells
- CCR6 and CXCR6 in Th17 cells
- CCR9 and 10 for T cells homing to the small and large intestine, respectively
- CCR7 for naïve T cells and central memory T cells; both home to lymph nodes

Other homing receptors are adhesion molecules.

- Antigen-experienced CD4 T cells can be induced to express α4β7 integrin through a retinoic acid-dependent mechanism ^15^.
- PSGL-1, a scaffolding glycoprotein that carries ligands for selectins. PSGL-1 is not functional on naïve T cells, but becomes functional upon induction of fucosyl transferase-VII; an enzyme highly expressed in Th1 cells ^16^.
- Cutaneous lymphocyte antigen (CLA) is a collection of glycoproteins that can serve as E-selectin ligands and drive homing of Th2 cells to the skin
- L-selectin (CD62L) is a homing receptor for naïve T cells and central memory T cells to home to lymph nodes

The Th1 chemokine receptor CXCR3 is induced soon after a naïve T cell sees antigen ^17^. Interestingly, induction of most homing receptors is T-bet dependent ^18^.

## T cell polarization

CD4 T cells differentiate to Th1, Th2, Th17, iTreg and TFH cells. All CD4 T cell expansion and activation is IL-2-dependent. It is useful to emphasize that IL-2 deficient mice suffer from autoimmune disease and not from immunodeficiency. The autoimmune phenotype is driven by a lack of regulatory T cells. This suggests that the primary function of T cell activation and expansion is the production of iTregs, which appears to be necessary because central tolerance (elimination of self-reactive T cell clones in the thymus) is ineffective ^19, 20^. Although T cell clones are eliminated in the thymus by negative selection, other T cell clones differentiate to natural regulatory T cells (nTregs). nTregs show chromatin remodeling that allows stable FoxP3 expression. nTregs are produced in the thymus and not in secondary lymphoid organs and thus are not subject to the second touch hypothesis and therefore not considered further.

- **iTreg**
  These induced regulatory T cells are mainly dependent on TGF-β1, a product of M2 macrophages and many tissue cells ^21^. The dominant role of TGF-β1 is demonstrated by lethal autoimmune disease in TGF-β1 knockout mice ^22^, which is phenocopied by T cell-specific disruption of TGF-β receptor II ^23^. iTregs express the transcription factor FoxP3. FoxP3 expression is induced by IL-27 and IFNγ through a STAT1-dependent mechanism, by TGF-β through SMAD3, TIEG and ITCH, and by retinoic acid (RA) through an unknown mechanism ^13, 24^. FoxP3 is inhibited by IL-27 through a STAT3-dependent mechanism, by IFN-γ through IRF1, by IL-4 through STAT6, by IL-5 or IL-6 through STAT5 and by S1P1, CD28 and IL-21 through unknown mechanisms. Under the influence of inflammatory cytokines like IL-6, iTregs can differentiate to Th17 ^25^.
- **Th17**
  These IL-17A- and IL-17F-producing CD4 T cells play central roles in host defense against fungal and some bacterial infections, but also in many autoimmune diseases. The defining transcription factor of Th17 cells is RORγt ( *Rorc*), but they also express RORα, IL-21 and IL-23R. Th17 cells are induced by IL-6 or IL-1 and TGF-β, but also IL-9 and IL-21 through a STAT3-dependent mechanism. Th17 cells are induced by segmented filamentous bacteria in the intestinal flora and by DCs activated by dectin-1 ligands. Th17 differentiation is inhibited by IL-27 (through STAT1) and by Th1- and Th2-driving cytokines. Some Th17 cells also express IL-10 ^26^.
- **Th1**
  These T-helper 1 cells secrete interferon-γ and TNF. Th1 differentiation is promoted by IL-12 through a STAT4-dependent signaling pathway and by IFN-γ through STAT1. The defining transcription factor is T-bet, encoded by the *Tbx21* gene. Tbx21 inhibits other directions of CD4 T cell differentiation.
- **Th2**
  T-helper 2 cells produce IL-4, IL-5 and IL-13. Although Th2 cells can develop in response to IL-4, IL-5 and IL-13, the Th2-inducing stimulus from antigen-presenting cells is not known. The defining transcription factor is GATA-3, encoded by the *Gata3* gene.
- **TFH**
  Follicular helper T cells develop in response to ICOS ligand and CD40. They migrate to B cell areas and provide essential help to B cells as they undergo differentiation and switch the antibody isotypes they secrete. The defining transcription factor is Bcl6.

CD4 T cells produce memory cells that can be divided into effector- (TEM), central memory- (TCM) ^27^, and into resident memory cells (TRM) that stay in the skin and intestinal epithelium ^28^. TEM are CD45RB ^low^, CD44 ^high^ and LFA-1 ^high^. TCM express CCR7 and CD62L. TRM express CD103 and CD69. It is currently controversial whether long-lived memory T cells originate from effector T cells (the cells expanding during the acute insult) or represent a separate lineage ^29^. There is some evidence that memory T cells retain memory of the phenotype (Th1, Th2, Th17) they had during the initial encounter of antigen.

## Antigen presentation in non-lymphoid tissues

Antigen presentation to antigen-experienced T cells in non-lymphoid tissues is not well studied. Only a handful of reports touch on this subject.

Flugel’s group tracked antigen-specific CD4 effector T cells in the mouse brain under conditions of experimental autoimmune encephalitis (EAE) ^30^. Although the antigen presenting cells (APCs) were not directly visualized, 35% of the T cells stopped and appeared to make an immunologic synapse suggested by polarized expression of LFA-1 and TCR. This was antigen-specific, because ovalbumin-specific T cells were only tethered after intrathecal injection of antigen. Carbone’s group studied memory T cell activation using reactivation by herpes simplex virus in dorsal root ganglia of mice ^31^. Although no imaging was performed, the key finding is that resident T cells can expand locally in an antigen-specific manner. Bousso’s group studied *Leishmania major* antigen recognition in the skin and found that CD4 T cells can activate nearby cells by IFN-γ, which can diffuse as far as 80 μm ^32^.

Steinman’s group isolated vascular dendritic cells from CD11c-YFP mouse aortas and aortic valves and incubated them with transgenic T cells ^33^. They found that these CD11c+ DCs expressed MHC-II, CD80 and CD86, but not CD40. These DCs were able to cross-present protein antigens on MHC-I to CD8 T cells. In a second study, the same group showed that these vascular DCs are dependent on the growth factor receptor Flt3, have tolerogenic properties and some of them express the integrin CD103 ^34^. In a study of antigen presentation in the atherosclerotic aorta, my lab showed that antigen-experienced (CD44 ^hi^CD62L ^-^), but not naïve CD4 T cells interact productively with APCs in the adventitia and the atherosclerotic plaque ^35^. The APCs were visualized by yellow fluorescent protein (YFP) driven by the CD11c promoter ^36^ and the T cells were labeled *ex vivo* to directly study interactions in the aorta. Three lines of evidence showed that the interactions were productive. First, long interactions between CD4 T cells and APCs were only observed when antigen was present. Second, the migration velocity of the T cells was drastically reduced during these interactions, from about 10 to about 3 μm/min. Third, cytokine production (IFN-γ, TNF) was observed when explanted aortas were incubated with antigen-experienced, but not naïve T cells. Taken together, these data show that a productive recall response is supported in the non-lymphoid tissue ^35^. We did not investigate which co-stimulatory molecules were involved in these interactions of CD4 T cells with APCs. We also did not identify the precise nature and phenotype of the DCs. Also, we did not retrieve the T cells from the aortas to study their commitment to Th1 or Th17 or other subsets. Finally, we did not study chromatin remodeling in these T cells.

The second touch hypothesis, if correct, allows several testable predictions:

- Incompletely committed T cells should be found in lymph nodes and/or in efferent lymph. These cells would express homing receptors like chemokine receptors and adhesion molecules, but would not be fully committed to T1, Th2, Th17 or Treg (i.e., lack epigenetic markers).
- T cell polarization would be expected to be more complete after interaction with APCs in non-lymphoid tissues. This could be tested by T-bet expression for Th1, RORγt expression for Th17 or FoxP3 expression for iTreg, and by looking for the epigenetic signatures of full commitment.
- A complete set of polarizing signals may be especially critical for iTregs, because they need to come in contact with active TGF-β produced by the same cell that presents the relevant antigen, and active TGF-β requires αVβ8 integrin expression on that same cell, which may be an M2 tissue-resident macrophage.
- Blocking CD28 or CD80 and CD86 in non-lymphoid tissues would be expected to only have a limited impact, because TNF superfamily molecules are expressed on APCs in non-lymphoid tissues and can serve as alternative co-stimulatory molecules.

An interesting example of CD4 T cell interaction with APCs in a non-lymphoid tissue is “licensing” of encephalitogenic T cells in the lung as detailed in ^37^. The details of this “licensing” process are not known. The licensing process is not antigen-specific.

## Chromatin remodeling and phenotypic stability

CD4 T-helper cells can interconvert between phenotypes. In general, iTregs and Th17 cells are thought to be less stable and Th1 and Th2 cells are thought to be more stable. A more stable phenotype is associated with chromatin remodeling in the region of key cytokine and transcription factor genes. This is best described for Th1 and Th2 cells.

The histone 3 lysine 4 trimethylation (H3K4me3) mark is associated with a permissive chromatin state, whereas H3K27me3 is suppressive. The H3K27me3 repressive tag is effectively removed by the enzyme encoded by *Jmjd3*, which is induced by T-bet, the defining transcription factor of Th1 cells. This includes removal of H3K27me3 from the *Ifng* locus ^38, 39^. To some extent, T-bet also represses genes that would be expressed in alternative T-helper fates, like Bcl6, the defining transcription factor of TFH cells ^18^. However, FOXP3 and T-bet can be co-expressed in the same CD4 T cell ^40^. Similarly, some FOXP3+ T cells secrete IL-17A ^41^. There is no accepted nomenclature for these “Th1 Tregs” and Th17 Tregs” yet.

The genes encoding the Th2 cytokines IL-4, IL-5 and IL-13 are found on mouse chromosome 11 very close to each other. This locus is extensively remodeled in Th2 cells. A 3’ enhancer of IL-4 expression called HSV is required for stable activation of the IL-4 promoter ^42^, especially in TFH cells. The epigenomic modifications leading to iTreg ^43^, Th17 and TFH are under investigation.

In the context of the second touch hypothesis, it would seem reasonable to postulate that the second touch is required for epigenomic modifications and stable T-helper phenotype commitment.

## Evidence supporting the second touch hypothesis

The finding that induction of most homing receptors is T-bet dependent ^18^ supports the hypothesis that acquisition of homing receptors may precede full T cell differentiation: T-bet is the defining transcription factor of the Th1 lineage, yet the homing T cells can be of any lineage. As a specific example, the Th1 chemokine receptor CXCR3 is induced rapidly, and the peak of its expression precedes maximal T cell expansion ^17^.

The expression profile of co-stimulating receptors (TNF superfamily members TNFSF and TNF receptor superfamily members TNFRSF) and cytokines (IL-12, IL-23, TGF-β) is different in macrophages and DCs in inflamed tissues compared to DCs in secondary lymphoid tissues ^44^. This finding supports the idea that the signals received by antigen-experienced CD4 T cells cruising through inflamed tissues are different from those received in secondary lymphoid organs. Myeloid cells in tissues are mostly macrophages, which are usually M2 polarized, secrete active TGF-β and thus promote Treg induction. In inflamed tissues, most macrophages are M1-polarized, secrete IL-12 and IL-23 and thus support Th1 and Th17 cell development.

Only a very small fraction of DCs are migratory. These few cells are unlikely to fully represent the entire cytokine environment of the inflamed tissue. Certainly, resident tissue macrophages, a cell type known to be M2 polarized and anti-inflammatory, are not migratory ^45^. It follows that it is likely that antigen-experienced T cells would encounter a different cytokine milieu and different co-stimulating receptors in the inflamed tissue itself rather than in the secondary lymphoid organs.

Tissue macrophages are M2-polarized and produce TGF-β. However, TGF-β is secreted in an inactive form and requires αVβ8 integrin for activation ^2– 4^. The activating integrin must be expressed on the same cell that presents the antigen, suggesting that only TGF-β secreted by αVβ8 integrin-expressing macrophages and DCs is relevant for iTreg induction. Therefore, only M2 resident macrophages (which cannot migrate to secondary lymphoid organs) in tissues would be expected to be effective at producing iTregs. There is good evidence that iTregs from non-lymphoid tissues are the ones that are relevant in curbing excessive inflammation ^13^. Therefore, one of the most striking consequences of the second touch hypothesis would be that only iTregs that circulate back into the tissue are effective at curbing inflammation, because it is only in the context of M2 tissue macrophages that they become effectively committed to the iTreg lineage.

In inflamed tissues, pro-inflammatory M1 macrophages, both converted from resting tissue macrophages and derived from newly recruited monocytes, express their own characteristic cytokines including IL-12 and IL-23. The vast majority of M1 macrophages remain in the inflamed tissue, but some leave and enter the draining lymph node. Under inflammatory conditions, the difference between dendritic cells and M1 macrophages blurs, because both are monocyte-derived ^46, 47^.

## Consequences of the second touch hypothesis

If the second touch hypothesis is correct, it follows that activated antigen-experienced CD4 T cells are “immature” effector T cells when they first leave the priming lymph node. They are immature, because they are not fully committed to a differentiation program yet. To fully commit, they would require the second touch of seeing antigen presented by APCs in the inflamed tissue, providing different co-stimulators, perhaps in the form of interactions between TNF superfamily members with their receptors, or via soluble cytokines, or both. The “immature” effector T cell may be on the path to differentiation, but may not have undergone chromatin remodeling, which is needed for stable T helper phenotypes.

In addition, the nature and location of the second touch should also influence memory T cell polarization. This is well documented in two cases. Memory cells derived from TFH cells have the phenotype of central memory (TCM) cells ^48^, because they reside in the secondary lymphoid organs and home back there (through CCR7, CD62L and other homing mechanisms). Conversely, effector T cells recirculating through an inflamed tissue (via CXCR3, CCR4, CCR9, α4β7 integrin or PSGL-1) would be expected to have a phenotype of effector memory T cells (TEM). They may show Th1, Th2 or perhaps Th17 polarization. Specifically, T cells that encountered antigen in the intestinal tract acquire expression of the α4β7 integrin and preferentially home back to the intestine ^15, 49^.

## Co-stimulation by TNF and TNF receptor superfamily members

**Figure 3.**
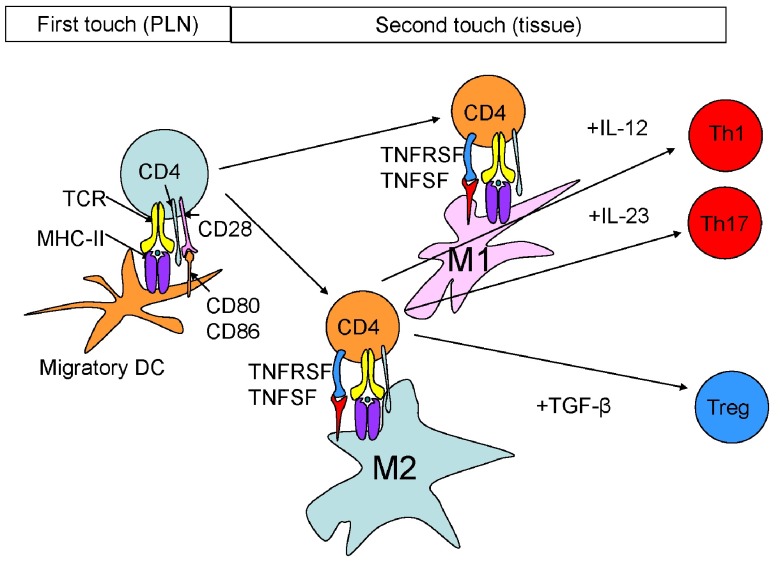
First and second touch. The first touch occurs in the lymph node, where the naïve T cell (light blue) is exposed to antigen (green circle) presented by MHC-II (purple) in the context of CD80 and CD86 (orange) co-stimulation, which bind CD28 (pink). The MHC-II-peptide complex is recognized by the T cell receptor (TCR, yellow) with CD4 (green). The second touch occurs in the tissue, where antigen is presented to antigen-experienced (orange) T cells by M1 (pink) and M2 (green) macrophages. Co-stimulatory molecules are likely from the TNFSF (red) on the T cell and the TNFRSF (blue) on the APC (see table for list of molecules). Characteristic M1 cytokines like IL-12 and IL-23 commit the CD4 T cell to Th1 and Th17, respectively. The M2 cytokine TGF-β commits T cells to the iTreg lineage.

Co-stimulation occurs not only through CD28 binding CD80 and CD86, but also through TNF and TNF receptor superfamily molecules on T cells binding their partners on APCs ^50^ ( Figure 3). The main co-stimulator molecules of this class are listed in Table 1.

**Table 1.** The gene names refer to the systematic names (TNF superfamily and TNF receptor superfamily).

Expressed by activated APC and activated T cells			Expressed by T cells		
Common name	CD	Gene	Common name	CD	Gene
OX40L	CD252	TNFSF4	OX40	CD134	TNFRSF4
4-1BBL		TNFSF9	4-1BB	CD137	TNFRSF9
	CD70	TNFSF7		CD27	TNFRSF7
TL1A		TNFSF15	DR3		TNFRSF25
TNF		TNFSF2	TNFR2	CD120b	TNFRSF1B
GITRL		TNFSF18	GITR	CD357	TNFRSF18

OX40, 4-1BB, CD27, and DR3 co-stimulatory molecules are constitutively expressed on Tregs and are either constitutive on conventional CD4 and CD8 T cells or induced usually within 24 hours after antigen presentation. Engagement of these receptors increases IL-4 and IFN- production, promotes division, and enhances survival. OX40 promotes effector T cells and blocks the development of iTregs. Inhibiting any of the listed pairs modifies many models of autoimmune disease. Taken together, the TNFSF and TNFRSF molecules are possible candidates for the second touch signal that T cells may receive in the inflamed tissue.

## Conclusion

In conclusion, the second touch hypothesis accommodates the plasticity of CD4 T cell phenotypes. It fills in the disconnect between the location of naïve T cell activation (PLN) and T cell effector functions (inflamed tissues). The concept of the second touch hypothesis generates many sub-hypotheses that can be tested by manipulating the mouse genome in a cell-specific fashion. Finally, the second touch hypothesis generates a conceptual framework for successful translation of immunological concepts to medical applications. This would include protective and tolerogenic vaccines and biologics that manipulate cytokines and their receptors.
