# Supplementary material for: The second touch hypothesis: T cell activation, homing and polarization
Source: F1000Res. 2014 Aug 4;3:37. Originally published 2014 Feb 5. [Version 2] doi: 10.12688/f1000research.3-37.v2 (PMC4038319; doi:10.12688/f1000research.3-37.v2)
Supplement: Peer review history

# Peer review: Referee response for version 2

**DOI:** 10.5256/f1000research.5174.r5691

**Published:** 2014-08-06

**Authors:** Lawrence P Kane

**Affiliations:** 1 Department of Immunology, University of Pittsburgh, Pittsburgh, PA, USA

The author has responded well to my comments. I think the paper is now acceptable for approval.

I have read this submission. I believe that I have an appropriate level of expertise to confirm that it is of an acceptable scientific standard.

# Peer review: Referee response for version 1

**DOI:** 10.5256/f1000research.3751.r4253

**Published:** 2014-04-14

**Authors:** Diana Boraschi

**Affiliations:** 1 Institute of Protein Biochemistry, National Research Council, Naples, Italy

In my opinion, this paper is important for two reasons. First, it builds on available data, looking in particular at discrepancies with the current theories, for building a new hypothesis that reconciles most of the unexplained results obtained in the past and were somehow forgotten or set aside. Secondly, by doing so it provides a very good example of how scientific thinking should work, a lesson that many young (and also less young) researchers need to learn. Research is going against dogmas, when dogmas do not explain real life, and formulating new hypotheses, which will the drivers of future research. In this particular case, the hypothesis that T cell polarisation/differentiation does not occur all at once, but that the terminal direction is reached in the specific site of the ongoing defense reaction is indeed a logical assumption, thus likely to be true. An important aspect of this paper is that the author puts the immune reaction right into the geographical and temporal framework of its development. Thus, where and when an immune response takes place could make a significant difference in its outcomes.

The paper is well written. The initial summary of current knowledge (which another reviewer, Lawrence Kane, has found too long) is indeed long, but not useless. It may be shortened but it is good to provide a clear picture of what is the current knowledge and hypothesis. Also, in full agreement with Lawrence’s comment, the author should emphasize the description of the second touch hypothesis, which in this version comes late and almost incidentally. I also agree on finding Table 1 confusing (I couldn’t even understand its title).

I am particularly pleased, as my major interest is in macrophages, that this hypothesis resurrects the role of macrophages as antigen-presenting cells, a role that has been for a long time forgotten with Steinman’s “marketing” of DC as the one and only APC. Macrophage polarisation into M1 and M2 is briefly touched in this paper, but this is OK since this was not a central issue. However, it may be worth remembering that M2 polarisation comprises, in the current terminology, all that is not M1, and therefore there are the inflammatory M2 macrophages that inform/support Th2 responses (alternative/type 2 inflammatory cells); the anti-inflammatory, tissue repair promoting cells (those that dump all types of inflammatory reactions, both type 1 and type 2, and produce matrix component, angiogenic and proliferation-inducing factors, etc.); and a heterogeneous group of intermediate functional phenotypes. Where exactly tissue resident macrophages fall in this description is still not clarified but since their reaction to any change in the tissue microenvironment is practically immediate, they will be functionally different depending on the tissue conditions - inflammation being a violent and massive kind of change in the tissue microenvironment.

I partly disagree with David Voehringer's comment that M1/M2 polarisation is dictated by Th1/Th2 cells, while Klaus in his paper state the reverse. Charles Mills *et al.* (2000) demonstrated how M1/M2 macrophages can actually direct T cell polarisation, opposite to the common belief that Th1/Th2 do regulate macrophage polarisation. I guess that the truth may be half way ( *in medio stat virtus*), since there are clear data that macrophages induce T cell polarisation and also data showing that macrophage polarisation is induced/amplified by polarised T cell cytokines. In a tissue during an ongoing inflammatory reaction, both things would have the possibility of occurring. We should also be aware that monocytes and lymphocytes infiltrating the site of inflammation do not come all at once at a single time point, but instead continuously during the entire course of the reaction - thus the antigen-experienced cells and the blood monocytes find, depending on when they arrive, completely different microenvironments and could therefore undergo different differentiation/regulation.

To conclude, I really liked the paper, not only its content but also its ability to stimulate my interest and wish to know more and go deeper.

I have read this submission. I believe that I have an appropriate level of expertise to confirm that it is of an acceptable scientific standard.

# Peer review: Referee response for version 1

**DOI:** 10.5256/f1000research.3751.r4188

**Published:** 2014-04-03

**Authors:** Luc Teyton

**Affiliations:** 1 Department of Immunology and Microbial Science, The Scripps Research Institute, La Jolla, CA, USA

I would make two major comments:

1. The title will be confusing for a lot of readers as it is reminiscent of the two signal hypothesis, which concerns naive T cell activation and the requirement for a co-stimulatory signal.
  Here, the issue is very different since the whole idea is that what happens to a T cell in the lymph node and in the inflamed tissue is substantially different. Here, everyone will agree, the set of effector functions that is displayed in the lymph node is meant to produce differentiation and proliferation. Whereas in inflamed tissue the effector functions will be the destruction of infected cells.
  In other words, the question could instead be asked as "Why do we have lymph nodes?”. Here, the discussion and hypothesis could go wild - but the concept of concentrating immune cells in a constrained space to optimize communications is very attractive.
2. Tissue versus lymph node - I agree with the author when he says that not enough is known about the trafficking of T cells to lymph nodes and tissues. Which comes first?
  It is counter-intuitive to follow the idea that priming of T cells is exclusively a lymph node process. Indeed, the molecular cues that allow T cells to enter tissues are coming from the tissue itself and the site of inflammation, not the lymph node. It is much easier to understand the story of the monocyte/dendritic cell if we consider the resident population - it will be at the site of injury and will receive the information to pickup antigen and migrate to lymph nodes.
  One thing that the author may want to consider in a revised version is the role of tissue anatomy in the circulation and recirculation of lymphocytes in tissues. Much of the ingress of lymphocytes into tissues has been studied from the capillary side. However, in tissues, lymphocytes are concentrated in the lymphatics, not in the capillary end of blood circulation. When taken into consideration, this factor appears essential for the priming in lymph nodes. In other words, what requires further study is the concentration of lymphocytes in tissue lymphatics and the demonstration that molecules such as TNF family members participate in the first phase of priming.

 

Minor comments: the description of the various T cell subsets (twice) is distracting from the important question - it should be simplified or simply referenced.

I have read this submission. I believe that I have an appropriate level of expertise to confirm that it is of an acceptable scientific standard, however I have significant reservations, as outlined above.

# Peer review: Referee response for version 1

**DOI:** 10.5256/f1000research.3751.r4184

**Published:** 2014-03-25

**Authors:** Lawrence P Kane

**Affiliations:** 1 Department of Immunology, University of Pittsburgh, Pittsburgh, PA, USA

This opinion piece by Ley is interesting and well-timed. Thus, the topic of peripheral T cell activation and differentiation is well-trodden ground at this point, but discussions of these events often stop at the border of the lymph node, where priming occurs. Here, Ley discusses the accumulating evidence that activated T cells require additional signals after their arrival in peripheral non-lymphoid tissues, in order to become fully differentiated. This is a topic that has received much less attention, and warrants further attention from T cell biologists.

I have three major suggestions for revision of the piece as it stands now:

1. The author dedicates approximately one-third of the article at the beginning to reviewing various aspects of T cell priming in lymph nodes. In my opinion, this runs the risk of losing the reader’s interest, before reaching the more interesting discussion of the “second touch” hypothesis. I would suggest significantly trimming this part of the article, since it has been so thoroughly reviewed elsewhere. In addition, the author could circle back to this after the more novel discussions of peripheral differentiation.
2. It would also be helpful to hear more about previous studies on TNF/TNFr family members and T cell differentiation, in the last section of the article (there should be more space if the first part is shortened, as suggested above).
3. Finally, I found the Table listing TNF/TNFr family members somewhat confusing. Does the heading “T cell” at the top refer to “ *constitutive expression on T cells*”?  The headings should be clarified or perhaps additional columns could be used for listing expression patterns.

 

Other, more minor, comments:

- Page 6, second column – CD28 is misspelled as “C28”.
- A few sentences could use additional editing to make them easier to read (e.g. page 6, first column has two such sentences).

I have read this submission. I believe that I have an appropriate level of expertise to confirm that it is of an acceptable scientific standard, however I have significant reservations, as outlined above.

# Peer review: Referee response for version 1

**DOI:** 10.5256/f1000research.3751.r4185

**Published:** 2014-03-25

**Authors:** David Voehringer

**Affiliations:** 1 Department of Infection Biology, Universitätsklinikum Erlangen and Friedrich-Alexander-Universität Erlangen-Nürnberg, Erlangen, Germany

This opinion articles provides a hypothesis that T cells are only weakly activated in lymphoid organs but required a "second touch" in inflamed tissues to stabilize their differentiation program into different T effector cell subsets.

The article is well written but would benefit from removing redundant information regarding the different T cell subsets and M1/M2 macrophages which are given at several places in the manuscript. Further, most published data indicate that M1/M2 macrophages are induced by Th1/Th2 cells rather than M1/M2 polarization being regulated by Th1/Th2 cells. The notion that basophils induce Th2 cells should be removed as recent reports do not support this finding.

I have read this submission. I believe that I have an appropriate level of expertise to confirm that it is of an acceptable scientific standard, however I have significant reservations, as outlined above.
